# Long-Term Outcome after Autologous Stem Cell Transplantation with Adequate Peripheral Blood Stem Cell Mobilization Using Plerixafor and G-CSF in Poor Mobilizer Lymphoma and Myeloma Patients

**DOI:** 10.1155/2011/517561

**Published:** 2011-11-29

**Authors:** Jan S. Moreb, Donya Salmasinia, Jack Hsu, Wei Hou, Christina Cline, Emma Rosenau

**Affiliations:** ^1^Department of Medicine, University of Florida, Gainesville, FL 32610, USA; ^2^Departments of Epidemiology and Health Policy Research, University of Florida, Gainesville, FL 32611, USA

## Abstract

Poor peripheral blood stem cell (PBSC) mobilization predicts worse outcome for myeloma and lymphoma patients post autologous stem cell transplant (ASCT). We hypothesize that PBSC harvest using plerixafor and G-CSF in poor mobilizers may improve long-term outcome. We retrospectively analyzed the data on patients who had second PBSC mobilization using plerixafor and G-CSF as a rescue. Nine lymphoma and 8 multiple myeloma (MM) patients received the drug. A control group of 25 MM and lymphoma patients who were good mobilizers with G-CSF only was used for comparison. Sixteen of the 17 poor mobilizers proceeded to ASCT, and one MM patient had tandem transplants. Length of hospital stay, infection incidence, granulocyte engraftment, and long-term hematopoietic recovery were not significantly different between the two groups. In conclusion, all poor mobilizers were able to obtain adequate stem cells transplant dose and had similar transplant course and long-term outcome to that of the control good mobilizers group.

## 1. Introduction

The use of peripheral blood stem cells (PBSCs) for autologous and allogeneic transplantation has increased significantly in recent years. According to the Center for International Blood and Marrow Transplant Research (CIBMTR) [1], more than 95% of autologous stem cell transplants (ASCTs) and more than 70% of allogeneic stem cell transplants are carried out with mobilized PBSC. The advantages of using PBSC over bone marrow include shorter engraftment time, less transfusions, shorter hospital stay, convenience of stem collection, and rapid restoration of the immune system [2–5].

The optimal PBSC mobilization strategy and the precise identification of patients at risk for poor mobilization need to be further studied. Traditionally, mobilization of PBSC for ASCT has been accomplished using cytokines alone or in combination with chemotherapy [6–8]. However, a significant proportion of lymphoma and multiple myeloma patients are poor mobilizers, that is, unable to achieve the minimal target cell dose during their first round of mobilization and require a second round of mobilization using salvage regimens. Studies have shown that there are still significant mobilization failures after these salvage regimens, in addition to added toxicity, morbidity, and increased cost [6, 7, 9, 10]. These patients face some serious consequences such as inability to undergo potentially curative autologous stem cell transplantation (ASCT), slow recovery of blood counts after autografting, and higher rate of relapse [11–14].

Plerixafor (Mozobil), formerly known as AMD3100 (Genzyme, Cambridge, Mass, USA), is a CXCR4 antagonist which has been recently approved for PBSC mobilization in multiple myeloma (MM) and non-Hodgkin's lymphoma patients (NHL) undergoing ASCT. At our institution, we participated in the pivotal phase III studies [15, 16] as well as treated patients with plerixafor on the compassionate use protocol. In this paper, we analyze the data on lymphoma and MM patients who received the drug as a rescue during a second cycle of mobilization using plerixafor and G-CSF. Because of the known effects of poor mobilization on engraftment and long-term outcome after ASCT, we hypothesized that better PBSC yields after mobilization with plerixafor and G-CSF may improve the speed of recovery of blood counts, reduce hospitalization days, and improve the long-term outcomes. In order to test our hypothesis, we retrospectively compared our poor mobilizers' characteristics and outcome with a similar group of patients who were successfully mobilized with G-CSF only (good mobilizers) and underwent ASCT during the same time period.

## 2. Patients and Methods

### 2.1. Study Design and Patients

This is a retrospective institutional review board approved study involving MM and lymphoma patients who underwent PBSC mobilization for ASCT. Patients who received plerixafor were identified through the records of our Clinical Trials Office. Total of 8 MM and 9 lymphoma (8 NHL and 1 HD) patients received plerixafor as a rescue mobilization in the Compassionate Use Protocol (CUP). All patients signed informed consents at the time of enrollment. Most likely, these patients were included in the publication by Calandra et al. [17]. As described before [17], entry into the protocol was limited to patients who had previously failed to proceed to apheresis due to low peripheral blood (PB) CD34+ cell counts (usually 10 cells per mL or less) or based on apheresis yield were unlikely to collect the minimum number for a single transplant, usually 2 × 10^6^ CD34+ cells per kg. In almost all cases this assessment was made from the first apheresis following the mobilization. All our CUP patients had previously failed to collect the minimal CD34+ cell dose (≥2.0 × 10^6^ CD34+ cells/kg) for single transplant or double that for tandem ASCT with G-CSF alone. Inclusion criteria included age of 18 to 70 years, failure of prior mobilization or collection, ability to undergo transplant, WBC count >3.0 × 10^9^ per liter, ANC >1.5 × 10^9^ per liter, PLT count >100 × 10^9^ per liter, serum creatinine ≤1.5 mg/dL, liver function tests within 2x upper limit of normal, Eastern Cooperative Oncology Group performance status of 0 or 1, recovery from acute toxic effects of prior chemotherapy, left ventricle ejection fraction >45%, Forced Expiratory Volume in the first second >60% of predicted or carbon monoxide diffusing capacity ≥45% of predicted, and negative test for HIV infection. Exclusions included brain metastases, acute infection, active infection with hepatitis B or C, fever (>38°C/100.4°F), hypercalcemia (>1 mg/dL above the upper limit of normal), and pregnancy.

The control group included similar patients who were good PBSC mobilizers and were picked based on their known disease diagnosis from a list of good mobilizers patients provided by the Stem Cell Laboratory. Good mobilizers were defined as patients who had received G-CSF alone as the mobilization regimen and achieved the minimal target cell dose of ≥2 × 10^6^ CD34+/kg for one transplant or ≥4 × 10^6^ CD34+ cells/kg for two transplants (MM patients) during their first mobilization cycle. Total of 10 MM and 15 lymphoma (14 NHL and 1 HD) patients were included in the control group (referred to as good mobilizers). These patients had already underwent ASCT during the same time period like the study group and had similar eligibility criteria for ASCT.

### 2.2. Administration of G-CSF and Plerixafor

The patients were given G-CSF as per site preference, and so our patients typically had two subcutaneous doses of G-CSF for a total of 10 *μ*g/kg, or ≥20 *μ*g/kg if they were deemed to be at high risk for poor mobilization, for 4 days. At approximately 2200 on the fourth day of treatment they were given a subcutaneous dose of 0.24 mg/kg plerixafor. On the morning of the fifth day, G-CSF was administered and apheresis (blood volumes as per site preference) began at approximately 10 h after the plerixafor dose. Administration of G-CSF, apheresis and administration of plerixafor were repeated daily until the patient collected sufficient cells for transplantation (minimum 2 × 10^6^ CD34+ cells per kg). Treatment was discontinued at the investigator's discretion if the patient failed to collect enough cells to warrant continuation. The number of CD34+ cells collected during each apheresis session was recorded.

### 2.3. Data Collected and Analyzed

**Patients' Characteristics**

Include age, sex, diagnosis, stage of disease, bone marrow involvement, number of prior chemotherapy regimens, prior radiation therapy, prognostic factors, time from diagnosis to PBSC mobilization, and number of ASCT.

**Stem Cell Mobilization Data**

Include mobilization regimen, G-CSF dose, number of apheresis days, white blood cell (WBC) and platelets (PLT) counts on day 1 of apheresis, CD34+ cell yield, CFU-GM counts in day 1 apheresis product.

**Transplant and Post-ASCT Data**

Include CD34+ transplant cell dose, time to granulocyte and PLT engraftment, length of hospital stay, rate of infections, graft durability, progression-free (PFS), and overall survival (OS). Engraftment of granulocytes was defined as the first of 3 consecutive days with absolute granulocyte count (AGC) ≥0.5 × 10^9^/L, while the PLT engraftment was defined as the first day where the PLT ≥20 × 10^9^/L without transfusions. Graft durability was defined as maintenance of normal (acceptable) blood counts at 3, 6, and 12 months after ASCT.

### 2.4. Statistical Analysis

The PFS and OS were estimated by the Kaplan-Meier method. Both PFS and OS were reported for MM and lymphoma poor mobilizer patients. The statistical significance of differences between the groups was evaluated using the unpaired *t*-test and calculating two-tailed *P* value. All statistical analysis was performed using the GraphPad software Prism 4 (San Diego, Calif).

## 3. Results

### 3.1. Patient Characteristics

We studied total of 18 MM and 24 lymphoma patients, including patients who received plerixafor (poor mobilizers) and those who were successfully mobilized without plerixafor (good mobilizers) who served as controls for comparison (see Tables 1 and 2). Overall, no major differences were noticed between the good and poor mobilizers with either MM or lymphoma diagnosis. It is important to point out that there was one Hodgkin's lymphoma patient in each group. The pretransplant conditioning regimen for lymphoma patients included busulfan 0.75 mg/kg PO or IV q 6 h for 16 doses on days −8, −7, −6, and −5 (total busulfan dose 12 mg/kg) combined with etoposide 10 mg/kg IV on days −4, −3, and −2 (total etoposide 30 mg/kg) and cyclophosphamide 60 mg/kg IV on days −3 and −2 (total cyclophosphamide 120 mg/kg) [11], and etoposide was omitted for patients who were ≥65 years old. The pre-transplant conditioning regimen for MM patients was intravenous melphalan 200 mg/m^2^ in most patients except for 3 in the poor mobilizers group and 2 in the good mobilizers group who had busulfan/cyclophosphamide ± etoposide, as published before [18].

Our institutional policy is to avoid weekend apheresis, and therefore chemotherapy + G-CSF mobilization is not usually used. Our standard mobilization regimen involves the use of G-CSF 5 *μ*g/kg BID. Patients with risk factors for poor mobilization will usually receive higher dose of G-CSF (10 or 16 *μ*g/kg BID). Indeed, and regardless of their final group designation, large number of MM patients (37.5–50%) and the majority of lymphoma patients (66.7–92.3%) received the high G-CSF dose for their first apheresis attempt (Table 3). In general, patients who were poor mobilizers and needed plerixafor for their second apheresis attempt received the same G-CSF dose that was used for their first mobilization. Thus many poor mobilizers ended up receiving high dose G-CSF with their plerixafor. The G-CSF dose was not specified in the CUP, and therefore, the use of the high G-CSF dose did not constitute a violation of the protocol. Plerixafor was given at the recommended standard dose of 0.24 mg/kg of body weight by subcutaneous injection at 2200 the day prior to each apheresis, that is, 10-11 hr before the start of apheresis.

### 3.2. Apheresis Outcomes

In order to study the effects of plerixafor on the apheresis and the quality of the stem cell product, we compared the study group to the control group of good mobilizers using the number of CD34+ cells collected, number of days of apheresis and the yield of CFU-GM in the product of the first apheresis, day for each patient (see Table 4). As expected, the median CD34+ cells/kg was slightly higher while the median number of apheresis days was lower in the good mobilizers. However, the quality of the collected stem cell product as measured by the number of CFU-GM colonies was similar in both plerixafor + G-CSF and G-CSF mobilized groups.

One myeloma patient in the plerixafor-mobilized patients with manual differential count on day 1 had 21% blasts and young cells in peripheral blood and 12% in the apheresis product. This MM patient from the poor mobilizers group relapsed with circulating plasma cells shortly after the apheresis, and flow cytometry analysis confirmed the presence of myeloma cells in his stem cell product. The patient did not undergo ASCT and died from his disease. In retrospect, the patient was already in relapse at the time of his 2nd mobilization and apheresis.

We further analyzed the 13 patients (4 MM and 9 lymphoma patients) who were truly poor mobilizers and failed to collect ≥2 × 10^6^ CD34+ cells/kg during the first mobilization with G-CSF only, thus excluding the myeloma patients who were defined as poor mobilizers based on their failure to collect ≥4 × 10^6^ CD34+ cells/kg for two transplants. We compared the 1st and 2nd stem cell mobilizations (Table 5). The results show that during the 2nd mobilization with plerixafor and G-CSF, higher WBC (*P* = 0.0003) was achieved on 1st day of apheresis. In addition, the amount of CD34+ cells/kg was significantly higher during the 2nd mobilization (*P* = 0.025). These results confirm the effectiveness of adding plerixafor to G-CSF. Indeed, with the combination of products from first and second mobilization cycles, all patients had adequate CD34+ cells dose for one or two ASCTs, although some of them achieved only the minimal doses required for single ASCT (2 × 10^6^ CD34+/kg). The median infused transplant CD34+ cell dose/kg for each group was as follows: MM poor mobilizers 2.96 × 10^6^ (range, 2.81–6.56 × 10^6^); MM good mobilizers 6.3 × 10^6^ (range, 4.61–8.9 × 10^6^); lymphoma poor mobilizers 4.69 × 10^6^ (range, 1.91–6.9 × 10^6^); lymphoma good mobilizers 6.64 × 10^6^ (range, 5.02–16.7 × 10^6^). 

### 3.3. Short- and Long-Term Hematologic Recovery

In order to evaluate the quality of the plerixafor mobilized stem cell products, we compared the study group to the good mobilizers group for different parameters such as length of hospital stay, infections during hospital stay, engraftment of granulocytes and platelets, and one-year hematopoietic recovery as reflected by normal (acceptable) blood counts.  Table 6 shows the hospital course and the time to engraftment were comparable between the good mobilizers and the poor mobilizers. Although in the lymphoma poor mobilizers group the median time to PLT engraftment (>20 × 10^9^/L) was 8 days longer than that of the good mobilizers; however, this difference did not reach a statistical significance.

The effect of stem cell mobilization on normalization of PB counts, WBC, hemoglobin (Hgb) and hematocrit (HCT), and PLTs, for both groups and diseases, was recorded. Again, at 12 months after ASCT, there was no significant difference between those who received plerixafor + G-CSF in the poor mobilizers group and those who received only G-CSF mobilization in the good mobilizers group. At 12 months after ASCT, the median values for WBC, Hgb, and PLTs were calculated for both poor and good mobilizers: for MM patients, median WBC 4.2 × 10^9^/L (range, 2.8–6.1 × 10^9^) and 5.1 × 10^9^/L (range, 3.6–9.0 × 10^9^), median Hgb 12.8 gr/dL (range, 10.8–15.4) and 12.3 gr/dL (range 10.3–14.1), and median PLTs 149 × 10^9^/L (range, 96–266 × 10^9^) and 192 × 10^9^/L (range, 150–297 × 10^9^), for poor and good mobilizers, respectively; for lymphoma patients, median WBC 5.1 × 10^9^/L (range, 2.2–11.8 × 10^9^/L) and 5.3 × 10^9^/L (range, 2.7–9.7 × 10^9^/L), median Hgb 11.6 gr/dL (range, 8.2–14.6) and 13.4 gr/dL (range, 7.1–15.5), and PLTs 125 × 10^9^/L (range, 37–304 × 10^9^) and 214 × 10^9^/L (range, 25–337 × 10^9^), for poor and good mobilizers, respectively. Statistical analysis to detect significant differences between the two groups within the lymphoma and myeloma patient populations was performed using the nonparametric Student's *t*-test. No significant differences were detected with the two-tailed *P* value being ≥0.266. There were similar proportions of lymphoma patients in both groups (22.3 versus 23.08%, poor versus good mobilizers, resp.) with at least one abnormal count, most likely reflecting the longer course of pre-ASCT chemotherapy that they usually receive in comparison to myeloma patients.

### 3.4. Long-Term Survival Outcomes

**Figure 1:**
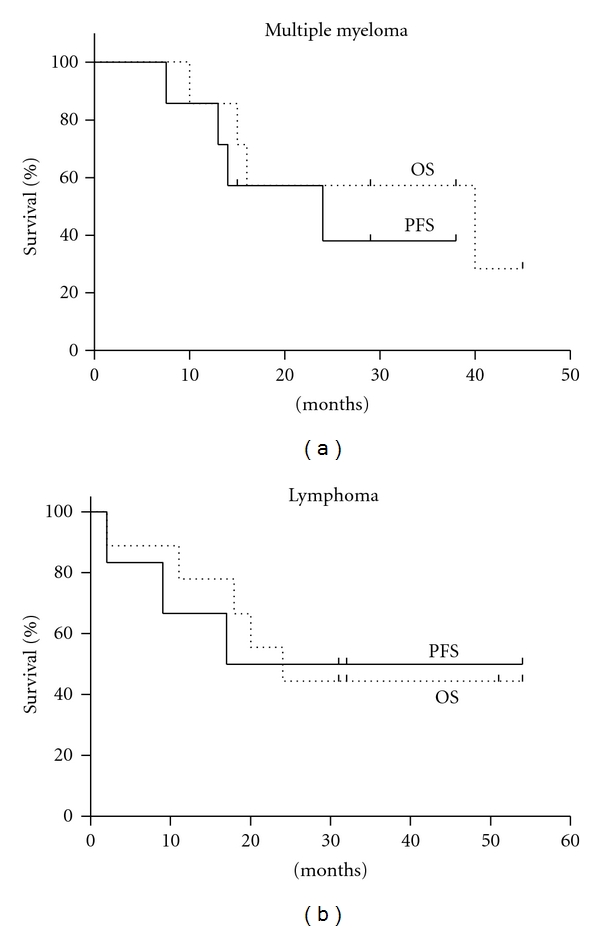
The Kaplan-Meier curves for PFS (solid line) and OS (broken line) for MM patients (a) and lymphoma patients (b). The data shown is only for the poor mobilizer patients.

We assessed the effects on relapse and survival of better mobilization with the use plerixafor. The Kaplan-Meier analysis for PFS and OS in the MM and lymphoma poor mobilizer patients is shown in Figure 1. Due to the retrospective nature of the study and the small number of patients, we refrained from performing a comparison to the control group.

In MM, the median followup for the poor mobilizers was 29 months (range, 10–45) and for the good mobilizers was 48.5 months (range, 11–58). For poor mobilizers, the median PFS and OS were 22.5 and 40 months, respectively. There was one relapse in each group during the first year after ASCT at 7.5 and 8 months.

In lymphoma patients, the median followup was 24 months (range, 2–54) for the poor mobilizers and 28 months (range, 0.6–55) for the good mobilizer control group. For the poor mobilizers, the median PFS and OS were 17 and 24 months, respectively. There were 3 relapses within the first year, between 3–9 months after ASCT in the poor mobilizers group, and 3 relapses in the good mobilizers group between 4–7 months after ASCT.

## 4. Discussion

The addition of plerixafor to G-CSF as first line regimen for peripheral blood stem cell (PBSC) mobilization has been shown to be safe and effective in two phase III placebo-controlled randomized studies in MM and NHL patients undergoing ASCT [15, 16] as well as in multiple phase II and retrospective studies in hard-to-mobilize patients [7, 14, 17, 19–21]. Plerixafor has been offered to patients who failed prior mobilization attempts on a compassionate use protocol (CUP) by Genzyme. The European compassionate use data has been recently published [19]. Total of 56 patients from 15 centers in Spain and the UK were analyzed. The results showed that 75% of patients collected ≥2 × 10^6^ CD34+/kg and that 63% underwent transplant. All patients engrafted neutrophils and PLTs. Followup was up to 6 months during which 3 patients died from disease progression (2) or viral pneumonitis (1). Five patients failed to mobilize adequately with plerixafor and G-CSF and were not able to proceed to ASCT. Two other publications from the CUP study showed success rates for plerixafor plus G-CSF of 66–85% [17, 20, 21].

In our study, all patients except one relapsed MM patient were able to undergo ASCT including two MM patients who underwent tandem transplants. The explanation for that could be due to the fact that in many of our patients, the G-CSF dose given with plerixafor was double the standard dose of 10 *μ*g/kg/day. The reason for the use of such high dose of G-CSF up front, especially in most of the lymphoma patients, is most likely related to individual preferences by the treating physicians as well as the use of higher G-CSF doses in patients with known risks for poor mobilization. We have previously reported poor PBSC mobilization to be as high as 52% in lymphoma patients [10].

In this study, we used good mobilizers group in order to compare the quality and long-term outcomes of PBSC mobilization using plerixafor plus G-CSF versus G-CSF alone. Several quality measures were compared including median number of CD34+ cells/kg collected, median number of CFU-GM/kg, median hospital stay, incidence of infections, time to engraftment of granulocytes and platelets, and evidence of tumor mobilization by peripheral smear. There were no significant differences between the study group and the control group in any of these measures. As expected and according to published studies, time to platelet recovery (≥20 × 10^9^/L) was slower in the poor mobilizers lymphoma patients (24 days) in comparison to the good mobilizers group (16 days), but this was not statistically significant. Overall, all patients engrafted as expected and there were no cases of primary or secondary graft failures up to 12 months after the transplant. There were no treatment-related deaths in the plerixafor-mobilized patients, while two patients in the good mobilizers group died early from transplant complications.

In this study, one myeloma patient who was remobilized using plerixafor while he was already in relapse, developed full blown relapse shortly after the stem collection and died from plasma cell leukemia. Thus, caution should be exercised in the use of such powerful mobilizing combination of plerixafor and G-CSF in relapsing MM patients. Examining the PFS curves for the myeloma poor mobilizer group, there was no early relapses to suggest possible myeloma mobilization and reinfusion of myeloma contaminated stem cell products. On the other hand, there were early relapses in the plerixafor-mobilized lymphoma patients, but that may be reflective of the known higher risk for relapse in poor mobilizer lymphoma patients rather than reinfusion of lymphoma contaminated stem cell products.

In the truly poor mobilizers patients (failed to achieve ≥2 × 10^6^ CD34+ cells/kg in first mobilization cycle) who received plerixafor for their second mobilization, we have seen significant increases in the WBC counts on the 1st day of apheresis in comparison to those seen in the first mobilization cycle. These patients also had one less day of apheresis with significant increase in the median CD34+ cells/kg collected. These findings strongly support the published data that show plerixafor to be a very effective and well-tolerated agent [14–17, 19–28]. Furthermore, the success rate of plerixafor plus G-CSF appears superior to those described previously with other alternative mobilization protocols [6, 7, 9, 29, 30]. Prior treatment with lenalidomide has been reported to increase the risk of poor mobilization significantly in MM patients [31, 32]. In our poor mobilizers group, two MM patients had prior lenalidomide (≥4 cycles) and were successfully mobilized using plerixafor for their second mobilization cycle. The effectiveness of plerixafor in MM patients previously treated with lenalidomide has been recently reported by Micallef et al. [33].

We and others have reported that poor mobilizer lymphoma and MM patients had worse PFS and OS in comparison to the good mobilizers [11–14]. Therefore, it is logical to use these end points to measure the impact of plerixafor plus G-CSF mobilization. If more stem cells are mobilized with the use of plerixafor in poor mobilizers that result in better and faster recovery of hematopoiesis, then it is possible that the overall survival of this subset of patients may improve. Unfortunately, our current study is not designed to provide such information because of the retrospective nature and small number of patients. In this paper, we provide the PFS and OS for the poor mobilizer group only. Future studies should be designed to determine if there is indeed a positive effect of plerixafor on PFS and OS of lymphoma and MM patients undergoing ASCT.

## 5. Conclusions

Our results confirm the effectiveness of plerixafor in mobilizing and harvesting PBSC from lymphoma and myeloma patients who failed G-CSF mobilization and therefore allowing more patients to undergo ASCT. In addition, our study also show comparable long-term engraftment and hospital course for these poor mobilized patients in comparison to control good mobilized group. 

## Figures and Tables

**Table 1:** Patient characteristics: MM patients.

	Poor mobilizers (*n* = 8)	Good mobilizers (*n* = 10)
Age, median (range), years	61 (48–67)	65.5 (30–73)

Sex		
Male Female	8 0	5 5

Disease stage		
II III	2 6	4 6

Disease status		
CR/VGPR PR DP	2 6 0*	3 7 0

B2M Chromosome 13q/13 del	2.74 (1.68–5.84) 2	3.09 (1.3–5.18) 4

Median no. of prior treatment regimens (range)	1 (1–3)	1 (1–3)

Prior lenalidomide (≥4 cycles)	2	0

Prior RT	2	6

Median time from diagnosis (range), days	206 (129–462)	204 (109–377)

Patients who had two transplants:		
As salvage As tandem	1 —	1 1

Posttransplant maintenance**	5	6

*One patient was in PR at first mobilization; however, he developed disease progression by the time he went through the plerixafor mobilization. The patient did not go through ASCT, and he was excluded from survival analysis.

**At 3 months of evaluation posttransplant, patients were offered maintenance therapy with thalidomide.

*Note*. The results show number of patients in each category unless indicated otherwise.

**Table 2:** Patient characteristics: lymphoma patients.

	Poor mobilizers (*n* = 9)	Good mobilizers (*n* = 15)
Age, median (range), years	60 (36–65)	62 (21–75)

Sex,		
Male Female	5 4	9 6

Disease stage		
II III IV	1 2 6	4 0 10

HD NHL	1 8	1 14

Disease status		
CR PR Relapse	6 2 1	11 4 0

Median no. prior treatment regimens (range)	2 (1–3)	2 (1–3)

Prior RT	6*	5

Median time from diagnosis (range), days	730 (376–1327)	1350 (72–6603)

BM involvement	2	0

*One patient had radiation therapy (RT) during childhood.

*Note.* The results show number of patients in each category unless indicated otherwise.

**Table 3:** The mobilization regimens used except when nonapplicable (N/A). Plerixafor dose was 0.24 mg/kg for all patients given at 2200 the night before apheresis.

	1st mobilization attempt			2nd mobilization attempt	
G-CSF dose	5 BID *μ*g/kg	10 BID *μ*g/kg	16 BID *μ*g/kg	5 BID *μ*g/kg	10 BID *μ*g/kg
MM, *n *					
Poor mobilizers (*n* = 8) Good mobilizers (*n* = 10)	5 4	3 5	0 1	5 N/A	3 N/A

Lymphoma, *n *					
Poor mobilizers (*n* = 9) Good mobilizers (*n* = 13)^∗‡^	3 1	6 12	0 0	2 N/A	7 N/A

*One lymphoma control patient had chemotherapy + G-CSF 10 *μ*g/kg twice per day (BID).

^‡^In this group of 15 control lymphoma patients, mobilization regimen doses were not available for two patients.

**Table 4:** Summary of apheresis yields in the different groups of patients.

	CD34+ ×10^6^/kg Median (range)	Median days of apheresis/cycle	Median no. of CFU-GM ×10^5^/kg*
MM			
Poor mobilizers (*n* = 8) Good mobilizers (*n* = 10)	8.38 (2.4–25.1) 10.1 (6.4–12.3)	4 (2–5) 2 (2-3)	5.15 (0.45–9.3) 3.87 (0.0–13.9)^†^

Lymphoma			
Poor Mobilizers (*n* = 9) Good Mobilizers (*n* = 15)	3.85 (1.17–8.98) 5.56 (2.1–14.7)	3 (1–4) 2 (2–4)	2.01 (0.66–6.53) 2.01 (0.42–10.6)

*These numbers are from day 1 collection.

^†^One patient had 0 CFU-GM in one CFU assay.

**Table 5:** Comparison of laboratory and clinical data of truly poor mobilizers (defined as not achieving ≥2 × 10^6^ CD34+ cells/kg with first mobilization) during their 1st and 2nd mobilizations.

	1st mobilization (G-CSF alone)	2nd mobilization (G-CSF + Plerixafor)
Median number of apheresis days (range)	3 (2–5)	2 (1–5)

Median CD34+ ×10^6^/kg collected (range)	0.77 (0.24–1.98)	2.89 (0.72–22.5)*

Median WBC on 1st day of apheresis, ×10^3^/mm^3^ (range)	30.6 (5–59.6)	52.8 (21.2–94.6)*

Median PLT count on 1st day of apheresis, ×10^3^/mm^3^ (range)	114 (39–365)	132 (44–265)

^†^The number of poor mobilizers was 13, 4 multiple myeloma (MM) and 9 lymphoma patients. One MM patient died before transplant, and two patients had tandem transplants. Median transplant cell dose for these patients was 3.68 × 10^6^ CD34+/kg (range, 1.92–5.01).

*Significantly different with *P* ≤ 0.025.

**Table 6:** Hospitalization course and engraftment: comparison between poor and good mobilizers.

	Median hospital stay (range)	Patients with proven infections during hospital stay, n *	Median days to AGC ≥ 500/mm^3^ (range)	Median days to PLT >20 × 10^3^/mm^3^ (range)
MM				
Poor mobilizers (*n* = 8) Good mobilizers (*n* = 10)	19 (15–25) 17 (16–24)	4 5	14 (12–15) 12 (10–15)	13 (11–46) 12.5 (10–20)

Lymphoma				
Poor mobilizers (*n* = 9) Good mobilizers (*n* = 15)	22 (19–52) 22 (18–30)	2 6	12 (11–33) 12.5 (10–21)**	24 (17–58) 16 (11–240)***

*Infections include bacteremias, pneumonia, clostridium difficile colitis, neutropenic colitis, and fungal infection.

**One patient died during engraftment.

***In addition to the patient who died during engraftment, one patient never reached platelet recovery until death.

NOTE: no significant statistical differences in any of the above categories between poor mobilizers and control good mobilizers.

## Abbreviations

AGC: Absolute granulocyte count
ASCT: Autologous stem cell transplant
BID: Twice daily
CIBMTR: Center for International Blood and Marrow Transplant Research
CUP: Compassionate use protocol
HD: Hodgkin's disease
MM: Multiple myeloma
NHL: Non-Hodgkin's lymphoma
OS: Overall survival
PB: Peripheral blood
PBSC: Peripheral blood stem cell
PFS: Progression-free survival.
